# Red cell distribution width is an independent factor for left ventricular diastolic dysfunction in patients with chronic kidney disease

**DOI:** 10.1007/s10157-014-1033-7

**Published:** 2014-09-25

**Authors:** Leszek Gromadziński, Beata Januszko-Giergielewicz, Piotr Pruszczyk

**Affiliations:** 1 Department of Internal Diseases, Gastroenterology and Hepatology, University Clinical Hospital in Olsztyn, ul. Warszawska 30, 10-082 Olsztyn, Poland; 2 Department of Internal Diseases, Gastroenterology, Cardiology and Infectiology, University of Warmia and Mazury in Olsztyn, Olsztyn, Poland; 3 Department of Internal Medicine and Cardiology, Medical University of Warsaw, Warsaw, Poland

**Keywords:** RDW, Chronic kidney disease, Left ventricular diastolic dysfunction

## Abstract

**Background:**

The increased value of the red cell distribution width (RDW) was reported to indicate poor prognosis in patients with chronic heart failure. We evaluated the value of the RDW in the diagnosis of left ventricular diastolic dysfunction (LVDD) in patients without diastolic heart failure among the chronic kidney disease (CKD) population.

**Methods:**

The study group consisted of 73 ambulatory patients with CKD, stages 2–5. Standard echocardiography and tissue Doppler imaging (TDI) were performed, and the level of RDW was determined. Patients were divided into four groups according to the results of peak early diastolic velocity of mitral annulus (EmLV) and the stage of CKD: group with early stage CKD (eGFR > 30 ml/min/1.73 m^2^) without LVDD (EmLV ≥ 8 cm/s), early stage CKD with LVDD (EmLV < 8 cm/s), group with advanced stage CKD (eGFR ≤ 30 ml/min/1.73 m^2^) without LVDD, and group with advanced stage CKD with LVDD.

**Results:**

Patients with advanced stage CKD with LVDD were characterized by higher RDW levels than patients with advanced stage CKD without LVDD and with early stage CKD groups with and without LVDD [14.5 (13.8–19.5) % vs. 13.7 (11.4–15,4) %, *p* = 0.049, vs. 13.8(13.1–14.9) %, *p* = 0.031, vs. 13.7(12.1–16.2) %, *p* = 0.0007], respectively. The area under the receiver operating characteristic (ROC) curve of RDW level for the detection of LVDD was 0.649, 95 % confidence interval (CI) 0.528–0.758, *p* = 0.021, whereas ROC derived RDW value of >13.5 % was characterized by a sensitivity of 83.3 % and specificity of 45.2 % for predicting LVDD. The only independent factor of LVDD was RDW level >13.5 % with odds ratio (OR) = 3.92 (95 % CI 1.05–14.56), *p* = 0.037.

**Conclusion:**

RDW can be used as an additional factor for the diagnosis of LVDD in patients with advanced stage of CKD.

## Introduction

Red cell distribution width (RDW) is a measurement of the size variability of the red blood cell population. It is assessed by standard blood count usually automatically, and is widely available [1]. Generally, a high RDW level may reflect reticulocytosis, hemolytic disorders [2]. It has also known that RDW levels are elevated in inflammatory bowel diseases, pregnancy, liver and kidney diseases, and during inflammatory processes [3–5]. Recently, the increased value of the RDW was reported to indicate poor prognosis in patients with chronic heart failure, coronary artery disease, and pulmonary hypertension [6–11]. It has also shown that the RDW is associated with endothelial dysfunction in patients with chronic kidney disease (CKD) [12]. This interest was spurred by the report from Celik A et al. [13] which showed that there is a strong, independent association between RDW and elevated left ventricular filling pressure (LVFP) among patients with diastolic heart failure (DHF). In the current study, we evaluated the value of the RDW in the diagnosis of left ventricular diastolic dysfunction (LVDD) in patients without DHF among the CKD population.

## Patients and methods

The study group consisted of 73 ambulatory patients with CKD, stages 2–5, with preserved LV systolic function defined by left ventricular ejection fraction (LVEF) >50 % and with sinus rhythm. Exclusion criteria comprised: non-sinus rhythm, LV systolic dysfunction, previous myocardial infarction, cardiomyopathy, severe valvular heart disease, pericardial fluid, active chronic inflammation, or acute infectious diseases within 4 weeks. Diagnostic criteria for CKD were consistent with the National Kidney Foundation Kidney Disease Outcomes Quality Initiative (KDOQI) standards [14].

### Echocardiography

Standard echocardiography was performed for all patients using a GE 6S device with 2.5–3.5 MHz transducer. To increase the credibility of the obtained echocardiographic results, the physician who performed the examination was unaware of the biochemical parameters of the patients. The examinations were conducted in stable patients and particular attention was placed on retaining optimal hydration.

Using the M-MODE in the parasternal long-axis view the following parameters were measured: left ventricular end-diastolic dimension (LVEDD), right ventricular end-diastolic dimension (RVEDD), left atrial diastolic dimension (LAD), interventricular septal diastolic diameter (IVSDd), and left ventricular LV posterior wall dimension at diastole (LVPWd). In a four chamber view, left ventricular ejection fraction (LVEF) was measured by modified Simpson’s method [15]. Left ventricular mass (LVM) was calculated by the formula described by Devereux et al., and left ventricular mass index (LVMI) was obtained by dividing the left ventricular mass by the body surface area [16]. To assess transmitral flow, pulsed wave Doppler echocardiography was performed in a four chamber view. The Doppler gate was placed at the tips of the mitral valve leaflets and a two-phase flow profile was obtained, including: early (E) and late (A) transmitral velocities, deceleration time (DT) of the E wave, E/A ratio was also calculated [15].

### Tissue Doppler echocardiography

Tissue Doppler parameters were measured: peak mitral annular systolic velocity (SmLV), peak early diastolic velocity (EmLV), and peak late diastolic velocity (AmLV) [17]. These parameters were obtained from the apical four chamber view. In pulsed wave tissue Doppler echocardiography, diastolic and systolic velocities were measured by placing the Doppler gate on the lateral mitral annulus at the posterior leaflet of the mitral valve. The ratio of peak early transmitral velocity to peak early diastolic velocity (E/Em) was calculated for the lateral annulus. All parameters were calculated as the mean of measurements taken in three consecutive cardiac cycles. LVDD was defined as EmLV < 8 cm/s [18].

### Biochemical tests

On the day of echocardiographic examination, the following laboratory parameters were measured for patients: serum creatinine concentration, eGFR evaluated by the modified MDRD formula, as well as the serum levels of urea, parathormone (PTH), C-reactive protein (CRP), serum levels of albumin, and NT-proBNP levels were calculated by immunoassay with the Stratus ^R^ CS Acute Care ™ Siemens.

Additionally, blood samples were taken and following parameters were recorded: hemoglobin concentration (Hb), hematocrit (Ht), mean corpuscular volume (MCV), platelets (PLT), red blood cells (RBC), mean corpuscular hemoglobin (MCH), mean corpuscular hemoglobin concentration (MCHC), red cell distribution width (RDW), mean platelet volume (MPV), platelet distribution width (PDW), plateletcrit (PCT), and platelet-large cell ratio (P-LCR).

Patients were divided into four groups depending on the results of eGFR level. Group 1 (eGFR 89–60 ml/min/1.73 m^2^), Group 2 (eGFR 59–30 ml/min/1.73 m^2^), Group 3 (eGFR 29–15 ml/min/1.73 m^2^), and Group 4 (eGFR < 15 ml/min/1.73 m^2^). Subsequently all patients were divided into another four groups according to the results of EmLV and the stage of CKD: early stage CKD (eGFR > 30 ml/min/1.73 m^2^) without LVDD (EmLV ≥ 8 cm/s), early stage CKD with LVDD (EmLV < 8 cm/s), advanced stage CKD (eGFR ≤ 30 ml/min/1.73 m^2^) without LVDD, and advanced stage CKD with LVDD.

### Statistical analysis

Values of parameters with a normal distribution were presented as a mean ± SD, whereas values with non-normal distributions were expressed as median and range. To compare four groups, analysis of variance (ANOVA) was used. Pearson’s or Spearmans’s correlation tests were used for correlation between variables. A receiver operating characteristic (ROC) analysis curves served to determine the optimal cut-off point of RDW and NT-proBNP for identifying patients with LVDD. Areas under the curve were calculated as measures of the accuracy of the tests. Multivariate logistic regression analysis was used to determine which factors are independently associated with EmLV < 8 cm/s. To assess the diagnostic value, odds ratio for particular laboratory and echocardiographic parameters was calculated. In the analysis, the parameters were treated either continuously or dichotomously using their values as determined in the ROC analysis. A value of *p* < 0.05 was considered statistically significant.

### Ethics approval

All patients consented in writing for the inclusion in the research. The study protocol was approved by the Bioethics Committee (no 555/2011).

## Results

The current study included 73 patients with CKD in stages 2–5 (28 male and 45 female with mean age 66.7 ± 13.3 years). CKD etiology in the study group included: hypertensive and ischemic nephropathy in 32 patients, glomerulonephritis in 5 patients, interstitial nephritis in 7 patients, polycystic kidney disease in 6 patients, autoimmune disease in 1 patient, whereas unknown etiology was present in 22 cases. According to the eGFR levels, patients were divided into four groups, Group 1 with eGFR levels 89–60 ml/min/1.73 m^2^ consisted of 21 patients, Group 2 with eGFR levels 59–30 ml/min/1.73 m^2^ included 31 patients, Group 3 with eGFR levels 29–15 ml/min/1.73 m^2^ consisted of 14 patients, and Group 4 with eGFR levels <15 ml/min/1.73 m^2^ consisted of seven patients. The groups were compared between regarding all the biochemical variables (Table 1) and echocardiographic parameters (Table 2).

**Table 1:** Biochemical characteristics of patients from four groups according to the eGFR levels

Parameter	Group 1 (*n* = 21)	Group 2 (*n* = 31)	Group 3 (*n* = 14)	Group 4 (*n* = 7)	*p* < 0.05
Age (years)	59 (45–87)	68 (31–86)	69.5 (48–90)	71 (53–84)	–
BMI (kg/m^2^)	27 (19–39)	28 (23–42)	28 (23–33)	24 (21–34)	2–4
Creatinine (mg/dL)	0.8 (0.7–1.1)	1.4 (1.0–2.0)	2.8 (2.0–3.9)	3.7 (3.4–6.1)	1–2, 1–3, 1–4, 2–3, 2–4, 3–4
eGFR (ml/min/1.73 m^2^)	74 (62–85)	41 (30–59)	21 (15–29)	12 (6–13)	1–2, 1–3, 1–4, 2–3, 2–4
Urea (mg/dL)	34 (19–57)	49 (20–82)	116 (53–163)	137 (85–204)	1–2, 1–3, 1–4, 2–3, 2–4
PTH (pg/ml)	47 (24–75)	54 (28–151)	167 (66–326)	224 (96–408)	1–3, 1–4, 2–3, 2–4
NT-proBNP (pg/ml)	106 (12–511)	149 (38–643)	518 (46–4,967)	705 (1,947)	1–3, 1–4, 2–3, 2–4
Hb (g/dL)	13.7 (11.9–16.6)	12.8 (9.9–15.5)	11.2 (7.7–14.2)	11.1 (9.9–13.1)	1–3, 1–4
HT (%)	40 (35–49)	38 (23–46)	34 (23–43)	34 (30–40)	1–3, 1–4
PLT (10^3^/uL)	227 ± 81	228 ± 69	229 ± 93	172 ± 33	–
RBC (10^6^/uL)	4.4 (3.7–5.3)	4.3 (3.1–5.3)	3.8 (2.6–4.9)	3.7 (3.4–4.6)	1–2, 2–3
MCV (fL)	92 ± 4.5	89 ± 3.7	93 ± 6.7	91 ± 5.7	2–3
MCH (pg)	31.2 ± 1.3	29.6 ± 1.3	30.4 ± 2.6	29.5 ± 1.9	1–2
MCHC (g/dL)	34 (32–37)	33 (32–35)	33 (30–35)	33 (32–33)	1–3, 1–4
RDW (%)	13.6 (12.1–14.9)	13.7 (12.7–16.2)	14.7 (13.1–19.5)	14.3 (11.4–16.2)	1–3, 2–3
MPV (fL)	10.9 (9.1–13.4)	11.2 (8.9–12.5)	11.1 (10.1–14.6)	12.2 (10–13.8)	–
PDW (fL)	12.6 (10.5–20.1)	13.7 (9.5–17.0)	13.4 (11.6–23.6)	15.1 (11.9–20.9)	–
PCT (%)	0.25 (0.06–0.44)	0.25 (0.1–0.4)	0.23 (0.18–0.5)	0.18 (0.15–0.25)	–
P-LCR (%)	32.5 ± 8.4	33.6 ± 7.9	36.4 ± 9.2	38.1 ± 10.4	–
CRP (mg/L)	2.0 (0.5–6.0)	3.0 (1.0–36.0)	4.1 (3.0–36.0)	6.0 (1.0–8.0)	1–3, 2–3
Albumin (g/dL)	3.93 (3.22–4.58)	3.86 (3.33–4.6)	3.71 (2.62–4.12)	3.86 (3.81–4.68)	–

*BMI* body mass index, *eGFR* estimated glomerular filtration rate, *PTH* parathormone, *NT*-proBNP N-terminal pro brain natriuretic peptide, *Hb* hemoglobin concentration, *HT* hematocrit, *PLT* platelets, *RBC* red blood cells, *MCV* mean corpuscular volume, *MCH* mean corpuscular hemoglobin, *MCHC* mean corpuscular hemoglobin concentration, *RDW* red cell distribution width, *MPV* mean platelet volume, *PDW* platelet distribution width, *PCT* plateletcrit, *P-LCR* platelet-large cell ratio, *CRP* C-reactive protein, *1* group 1, *2* group 2, *3* group 3, *4* group 4

**Table 2:** Standard echocardiography and tissue Doppler echocardiography parameters in four groups of patients according to the eGFR levels

Parameter	Group 1 (*n* = 21)	Group 2 (*n* = 31)	Group 3 (*n* = 14)	Group 4 (*n* = 7)	*p* < 0.05
LVEDD (cm)	4.3 (3.6–5.8)	4.6 (4.0–6.1)	4.5 (3.7–6.0)	4.8 (4.1–5.6)	–
RVEDD (cm)	2.7 (2.1–3.0)	2.7 (2.2–3.3)	2.7 (2.4–3.0)	2.8 (2.2–3.2)	–
LAD (cm)	3.9 ± 0.5	4.0 ± 0.5	4.1 ± 0.5	4.2 ± 0.5	**–**
LVEF (%)	61 (51–61)	60 (50–76)	58 (54–70)	58 (55–71)	**–**
LVMI (g/m^2^)	80 (62–210)	90 (58–166)	94 (60–198)	100 (98–136)	**–**
IVSDd (cm)	1.0 (0.7–1.4)	1.1 (0.9–1.5)	1.2 (1.0–1.5)	1.2 (1.–1.4)	1–3
LVPWd (cm)	1.0 (0.7–1.5)	1.1 (0.9–1.3)	1.2 (1.0–1.4)	1.2 (1.0–1.3)	1–3
E (cm/s)	68 (53–116)	68 (44–111)	54 (35–93)	63 (38–100)	1–3
A (cm/s)	73 (55–111)	79 (50–118)	80 (58–141)	82 (47–130)	–
DecT (msec)	220 ± 46	222 ± 47	253 ± 52	225 ± 71	–
E/A	0.9 (0.6–1.4)	0.9 (0.5–1.6)	0.6 (0.4–0.9)	0.6 (0.4–0.9)	1–3, 2–3
SmLV (cm/s)	8 (6–14)	7 (5–10)	8.5 (6–13)	9 (7–14)	2–4
EmLV (cm/s)	9.0 ± 3.1	8.5 ± 2.3	6.8 ± 2.7	8.0 ± 3.2	–
AmLV (cm/s)	10.2 ± 3.0	9.6 ± 2.0	10.8 ± 2.5	11.3 ± 2.6	–
Em/AmLV	0.9 (0.3–2.1)	0.9 (0.4–2.0)	0.6 (0.4–1.1)	0.5 (0.4–1.8)	1–3, 2–3
E/Em	7.5 (5.5–13.1)	8.8 (4.7–13.8)	8.7 (4.8–14.2)	7.6 (5.2–11.7)	**–**

*LVEDD* left ventricular end-diastolic dimension, *RVEDD* right ventricular end-diastolic dimension, *LAD* left atrial diastolic dimension, *LVEF* left ventricular ejection fraction, *LVMI* left ventricular mass index, *IVSDd* interventricular septal diastolic diameter, *LVPWd* left ventricular left ventricular posterior wall dimension at diastole, *E* early transmitral peak velocity, *A* late transmitral peak velocity, *DT* deceleration time, *E/A* ratio of early transmitral peak velocity to late transmitral peak velocity, *SmLV* peak mitral annular systolic velocity, *EmLV* peak early diastolic velocity, *AmLV* peak late diastolic velocity, *Em/AmLV* ratio of peak early diastolic velocity to peak late diastolic velocity, *E/Em* ratio of early transmitral peak velocity to mitral annular early diastolic velocity, *1* group 1, *2* group 2, *3* group 3, *4* group 4

There were no significant differences between all groups in age, PLT, MPV, PDW, PCT, and P-LCR. Level of creatinine, urea, PTH, and NT-proBNP increased in parallel with the severity of kidney dysfunction. Hemoglobin, serum hematocrit, and MCHC were higher in Group 1 compared with groups 3 and 4. RBC level was higher in Group 1 compared with Group 2 and in Group 2 compared with Group 3, level of MCV was lower in Group 2 compared with Group 3 and MCH level was higher in Group 1 compared with Group 2. Whereas, level of RDW was higher in Group 3 compared with groups 1 and 2, [14.7(13.1–19.5) % vs. 13.6(12.1–14.9) %, *p* = 0.003 and vs. 13.7(12.7–16.2) %, *p* = 0.05], respectively. BMI was lower in Group 4 compared with Group 2.

Among 73 patients enrolled in our study, we obtained data on the CRP levels and serum albumin levels of 53 patients only. Patients in Group 3 were characterized by higher CRP levels than subjects in groups 1 and 2, (4.1 (3.0–36.0) mg/L vs. 2.0 (0.5–6.0) mg/L and vs. 3.0 (1.0–36.0) mg/L), *p* = 0.005 and *p* = 0.037), respectively. Whereas, level of serum albumin did not differ between four groups. In echocardiographic examination, there were no significane between all groups in LVEDD, RVEDD, LAD, LVEF, LVMI, A, DecT, EmLV, AmLV, E/Em ratio. While, IVSDd and LVPWd were higher in Group 3 compared with Group 1 and E wave was lower in Group 3 compared with Group 1. The ratio of E/A was lower in Group 3 compared with groups 2 and 1. The ratio of Em/AmLV determinated by TDI was also lower in Group 3 compared with groups 2 and 1. Additionally, SmLV was higher in Group 4 compared with Group 2.

To evaluate the RDW level in patients with and without LVDD (defined as EmLV < 8 cm/s), patients were divided into four groups according to the results of EmLV and the stage of CKD: early stage CKD (eGFR > 30 ml/min/1.73 m^2^) without LVDD (EmLV ≥ 8 cm/s) consisted of 33 subjects, early stage CKD with LVDD (EmLV < 8 cm/s) consisted of 16 subjects, advanced stage CKD (eGFR ≤ 30 ml/min/1.73 m^2^) without LVDD consisted of 10 subjects, and advanced stage CKD with LVDD with 14 subjects (Tables 3, 4).

**Table 3:** Biochemical characteristics of patients from four groups according to the stage of CKD and EmLV

Parameter	Early stage CKD without LVDD (*n* = 33)	Early stage CKD with LVDD (*n* = 16)	Advanced stage CKD without LVDD (*n* = 10)	Advanced stage CKD with LVDD (*n* = 14)	*p* < 0.05
Age (years)	64.4 ± 13.1	69.8 ± 10.3	65.2 ± 13.6	69.5 ± 15.8	–
BMI (kg/m^2^)	29.1 ± 5.5	29.6 ± 6.2	27.1 ± 3.1	26.7 ± 3.7	–
Creatinine (mg/dL)	1.1 (0.7–2.0)	1.2 (0.7–1.5)	3.2 (1.6–4.3)	3.0 (2.0–6.3)	1–3, 1–4, 2–3, 2–4
eGFR (ml/min/1.73 m^2^)	52 (31–85)	56 (35–79)	16 (10–30)	21 (6–29)	1–3, 1–4, 2–3, 2–4
Urea (mg/dL)	47 (19–74)	44 (29–62)	101 (68–204)	120 (53–163)	1–3, 1–4, 2–3, 2–4
PTH (pg/ml)	48 (24–107)	50 (30–85)	175 (64–408)	157 (66–346)	1–3, 1–4, 2–3, 2–4
NT–proBNP (pg/ml)	96 (12–549)	191 (35–643)	520 (65–966)	518 (46–4,968)	1–3, 1–4, 2–4
Hb (g/dL)	13.4 ± 1.3	13.5 ± 1.7	10.6 ± 1.6	12.0 ± 1.8	1–3, 2–3, 2–4
HT (%)	39 (13–46)	41 (13–49)	35 (26–38)	36 (13–43)	1–3, 2–3
PLT (10^3^/uL)	225 (66–453)	219 (45–334)	208 (150–287)	180 (127–484)	–
RBC (10^6^/uL)	4.5 ± 0.5	4.5 ± 0.6	3.6 ± 0.5	4.0 ± 0.6	1–3, 2–3
MCV (fL)	89.6 ± 4.5	90.0 ± 4.0	92.2 ± 5.3	91.1 ± 6.9	–
MCH (pg)	30.2 ± 1.7	30.3 ± 1.1	30.1 ± 1.3	30.1 ± 2.8	–
MCHC (g/dL)	34 (32–37)	34 (33–36)	33 (30–35)	33 (30–35)	2–4
RDW (%)	13.7 (12.1–16.2)	13.8 (13.1–14.9)	13.7 (11.4–15.4)	14.5 (13.8–19.5)	1–4, 2–4, 3–4
MPV (fL)	11.0 ± 1.0	11.0 ± 1.1	11.1 ± 0.7	11.6 ± 1.4	–
PDW (fL)	13.6 (9.5–20.1)	13.0 (10.4–16.7)	13.4 (11.6–23.6)	13.6 (11.6–23.6)	–
PCT (%)	0.24 (0.07–0.44)	0.25 (0.06–0.37)	0.25 (0.18–0.31)	0.23 (0.15–0.50)	–
P-LCR (%)	33.2 ± 8.2	33.3 ± 8.3	33.7 ± 5.7	38.0 ± 11.0	–
CRP (mg/L)	2.0 (0.5–30.0)	2.6 (1.6–36.0)	3.1 (1.0–19.0)	6.0 (1.0–36.0)	1–4
Albumin (g/dL)	3.82 (3.22–4.60)	3.98 (3.30–4.58)	3.88 (2.62–4.12)	3.77 (2.75–4.68)	–

*CKD* chronic kidney disease, *LVDD* left ventricular diastolic dysfunction, *BMI* body mass index, *eGFR* estimated glomerular filtration rate, *PTH* parathormone, *NT-proBNP* N-terminal pro brain natriuretic peptide, *Hb* hemoglobin concentration, *HT* hematocrit, *PLT* platelets, *RBC* red blood cells, *MCV* mean corpuscular volume, *MCH* mean corpuscular hemoglobin, *MCHC* mean corpuscular hemoglobin concentration, *RDW* red cell distribution width, *MPV* mean platelet volume, *PDW* platelet distribution width, *PCT* plateletcrit, *P-LCR* platelet-large cell ratio, *CRP* C-reactive protein, *1* Early stage CKD without LVDD, *2* Early stage CKD with LVDD, *3* Advanced stage CKD without LVDD, *4* Advanced stage CKD with LVDD

**Table 4:** Standard echocardiography and tissue Doppler echocardiography parameters in four groups of patients according to the stage of CKD and EmLV

parameter	Early stage CKD without LVDD (*n* = 33)	Early stage CKD with LVDD (*n* = 16)	Advanced stage CKD without LVDD (*n* = 10)	Advanced stage CKD with LVDD (*n* = 14)	*p* < 0.05
LVEDD (cm)	4.5 ± 0.5	4.8 ± 0.7	4.6 ± 0.3	4.7 ± 0.7	**–**
RVEDD (cm)	2.7 (2.1–3.0)	2.8 (2.3–3.3)	2.7 (2.3–3.0)	2.8 (2.2–3.2)	1–2
LAD (cm)	3.9 ± 0.5	4.2 ± 0.5	4.0 ± 0.3	4.2 ± 0.6	–
LVEF (%)	63 (51–76)	59 (50–66)	58 (54–69)	58 (54–71)	1–2, 1–3
LVMI (g/m^2^)	83 (58–138)	107 (74–210)	97 (81–127)	99 (60–198)	1–2
IVSDd (cm)	1.0 (0.7–1.5)	1.15 (1.0–1.3)	1.1 (0.9–1.4)	1.2 (1.0–1.5)	1–2, 1–4
LVPWd (cm)	1.0 (0.7–1.3)	1.2 (0.9–1.5)	1.1 (1.0–1.3)	1.2 (1.0–1.4)	1–2, 1–4
E (cm/s)	73 ± 17	59 ± 12	70 ± 15	54 ± 17	1–2, 1–4
A (cm/s)	71 (54–105)	79 (63–111)	79 (47–118)	80 (55–141)	–
DecT (msec)	217 ± 41	230 ± 56	222 ± 52	255 ± 60	–
E/A	0.94 (0.65–1.42)	0.70 (0.53–1.17)	0.84 (0.56–1.65)	0.60 (0.42–1.24)	1–2, 1.4
SmLV (cm/s)	8 (5–14)	7 (6–9)	9 (7–13)	7 (6–14)	1–2, 2–3
EmLV (cm/s)	9 (8–17)	6 (3–7)	11 (8–13)	6 (3–7)	1–2, 1–4, 2–3, 3–4
AmLV (cm/s)	9 (7–17)	10 (6–13)	12 (6–16)	10 (7–14)	–
Em/AmLV	1.0 (0.5–2.1)	0.55 (0.33–1.16)	0.88 (0.56–2.0)	0.50 (0.37–0.77)	1–2, 1–4, 3–4
E/Em	7.4 (4.7–11.2)	9.7 (6.9–13.8)	6.1 (4.8–11.6)	9.1 (6.2–14.3)	1–2, 2–3

*CKD* chronic kidney disease, *LVDD* left ventricular diastolic dysfunction, *LVEDD* left ventricular end-diastolic dimension, *RVEDD* right ventricular end-diastolic dimension, *LAD* left atrial diastolic dimension, *LVEF* left ventricular ejection fraction, *LVMI* left ventricular mass index, *IVSDd* interventricular septal diastolic diameter, *LVPWd* left ventricular left ventricular posterior wall dimension at diastole, *E* early transmitral peak velocity, *A* late transmitral peak velocity, *DT* deceleration time, *E/A* ratio of early transmitral peak velocity to late transmitral peak velocity, *SmLV* peak mitral annular systolic velocity, *EmLV* peak early diastolic velocity, *AmLV* peak late diastolic velocity, *Em/AmLV* ratio of peak early diastolic velocity to peak late diastolic velocity, *E/Em* ratio of early transmitral peak velocity to mitral annular early diastolic velocity, *1* Early stage CKD without LVDD, *2* Early stage CKD with LVDD, *3* Advanced stage CKD without LVDD, *4* Advanced stage CKD with LVDD

Among the biochemical parameters, patients with advanced stage CKD with and without LVDD as compared to patients both groups of early stage CKD were characterized by significantly higher level of creatinine, urea, PTH, and by significantly lower eGFR levels. NT-proBNP level was higher in advanced stage CKD with LVDD group compared with early stage CKD group with and without LVDD, and was higher in group with advanced CKD without LVDD than group with early stage CKD without LVDD. Whereas, NT-proBNP level did not differ between groups with and without LVDD in both groups of early and advanced CKD. Hemoglobin, serum hematocrit, and RBC were lower in advanced stage CKD without LVDD compared with both groups of early stage CKD. Additionally, hemoglobin was lower in advanced stage CKD with LVDD compared with early stage CKD with LVDD. MCHC level was lower in advanced stage CKD with LVDD compared with early stage CKD with LVDD, while CRP level was higher in advanced stage CKD with LVDD compared with early stage CKD without LVDD.

Patients with advanced stage CKD with LVDD as compared with advanced stage CKD without LVDD were characterized by significantly higher level of RDW [14.5 (13.8–19.5) vs. 13.7 (11.4–15.4) %, *p* = 0.049], respectively. Additionally, level of RDW was higher in advanced stage CKD with LVDD compared with both groups of early stage CKD. There were no significant differences between all groups in age, BMI and levels of PLT, MCV, MCH, MPV, PDW, PCT, P-LCR, and albumin.

At echocardiography, patients with early stage CKD without LVDD compared with early and advanced stage CKD with LVDD were characterized by significantly lower level of IVSDd and LVPWd and higher level of E wave and ratio of E/A. Additionally, patients with early stage CKD without LVDD compared with early stage CKD with LVDD were characterized by higher levels of LVEF, SmLV, EmLV, Em/AmLV ratio and lower levels of RVEDD, LVMI, E/Em ratio. Additionally, subjects with advanced stage CKD with LVDD were characterized by lower levels of EmLV and Em/AmLV ratio than subjects with advanced stage CKD without LVDD. While, the levels of E/A ratio and E/Em ratio did not differ between both groups of advanced stage CKD. There were no significant differences between all groups in LVEDD, LAD, A wave, DecT of E wave, and AmLV.

### ROC analysis

**Fig. 1:**
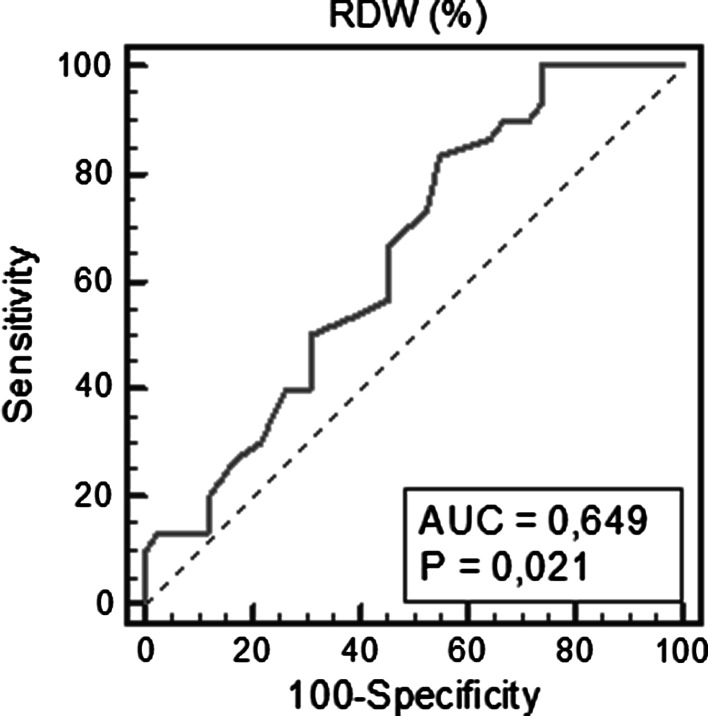
Receiver operating characteristic curves for RDW to predict LV diastolic dysfunction

The area under the ROC curve of RDW level for the detection of LVDD was 0.649, 95 % CI (0.528–0.758), *p* = 0.021. The optimal cut-off value in the ROC analysis for RDW was >13.5 %. This value was characterized by the sensitivity of 83.3 % for diagnosing LVDD and specificity of 45.2 %; positive predictive value (PPV) was 52 % and negative predictive value (NPV) was 79 % (Fig. 1).

**Fig. 2:**
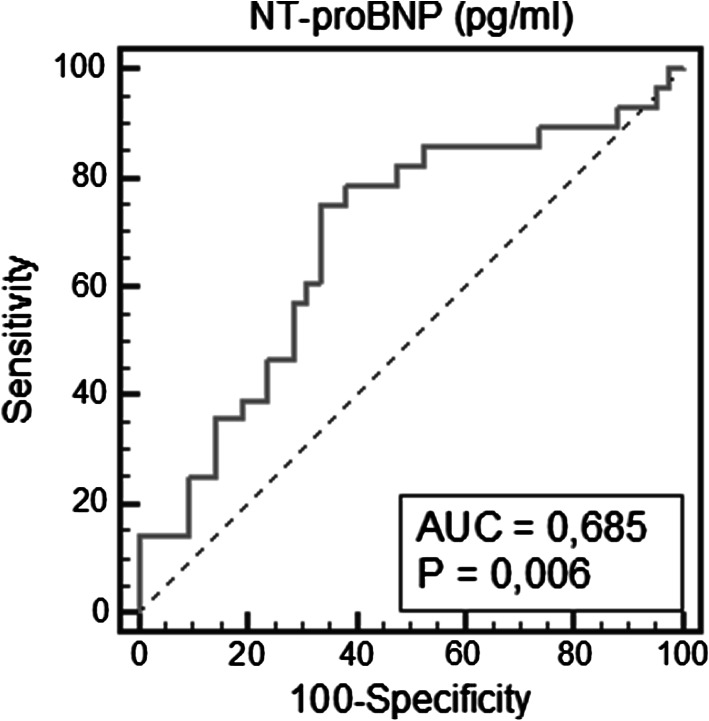
Receiver operating characteristic curves for NT-proBNP to predict LV diastolic dysfunction

The area under the ROC curve of NT-proBNP level for the detection of LVDD was 0.685, 95 %CI (0.563–0.791), *p* = 0.006. The optimal cut-off value in the ROC analysis for RDW was >171.2 pg/ml. This value was characterized by the sensitivity of 75.0 % and specificity of 66.7 % for diagnosing LVDD; PPV was 60 % and NPV was 80 % (Fig. 2).

There was also no significant differences for diagnostic accuracy between the two methods (*p* = 0.755).

### Correlation analysis

**Fig. 3:**
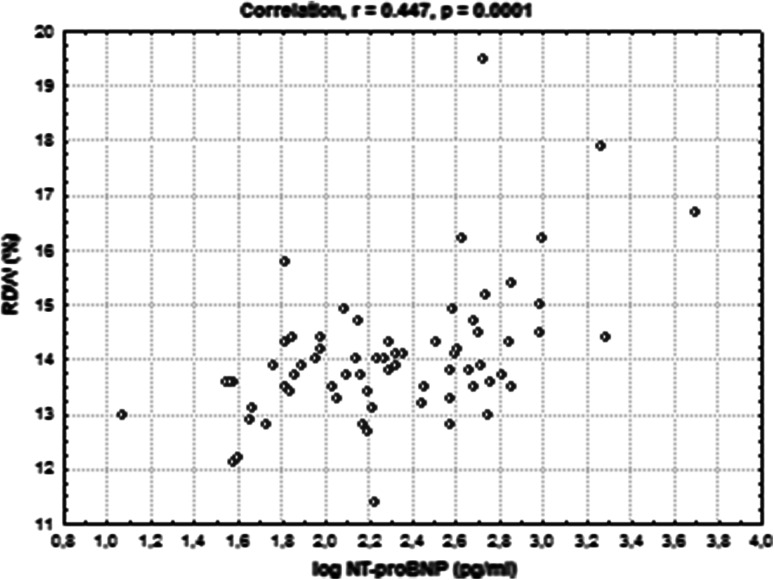
Positive correlation between RDW level and logNT-proBNP

**Fig. 4:**
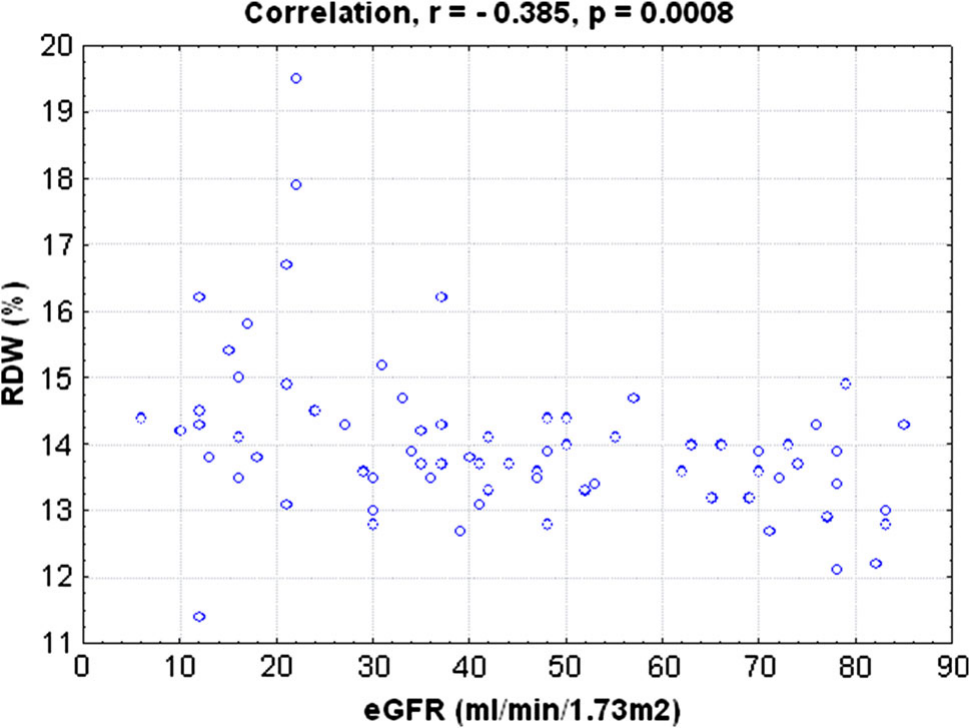
Negative correlation between RDW level and eGFR level

We investigated the correlations of RDW with laboratory and echocardiographic parameters that determine LVDD. RDW level was significantly correlated with log NT-proBNP (*r* = 0.447, *p* = 0.0001) (Fig. 3). We obtained also significant negative correlation between RDW level with level of eGFR (*r* = −0.385, *p* = 0.0008) (Fig. 4). Additionally, we performed the correlation between level of EmLV and following parameters: BMI, level of CRP, albumin, NT-proBNP, RDW (Table 5). Among these parameters, we obtained significant negative correlations between EmLV and CRP, NT-proBNP and RDW levels. Furthermore, we did not obtain the relationship between the antihypertensive treatment with use of inhibitors of rennin-angiotensin system and LVDD, *p* = 0.386.

**Table 5:** Correlation between EmLV levels and other factors

Parameters EmLV (cm/s)	*r*	*p*
BMI (kg/m^2^)	−0.124	0.293
CRP (mg/L)	−0.335	0.014
Albumin (g/dL)	0.052	0.713
NT-proBNP (pg/ml)	−0.305	0.010
RDW (%)	−0.234	0.047

*EmLV* peak early diastolic velocity, *r* Spearman correlation, *BMI* body mass index, *CRP* C-reactive protein, *NT-proBNP* N-terminal pro brain natriuretic peptide, *RDW* red cell distribution width

### Univariate and multivariate logistic regression

To determine the diagnostic value of laboratory parameters, univariate logistic regression analysis was performed and odds radio was calculated. Using stepwise regression, we created a model useful for the diagnosis of LVDD in CKD patients.

Only those parameters with *p* < 0.1 in univariate logistic regression were considered in multivariable analysis (Table 6).

**Table 6:** Biochemical parameters for the prediction of LV diastolic dysfunction (EmLV < 8 cm/s). Univariate and multivariate logistic regression analysis

Parameter	Univariate analysis			Multivariate analysis		
	OR	95 % CI	*p*	OR	95 % CI	*p*
Creatinine (mg/dL)	1.46	0.93–2.30	0.093	1.19	0.35–4.04	0.777
eGFR (ml/min/1.73 m^2^)	0.98	0.96–1.00	0.158			
Urea (mg/dL)	1.01	0.99–1.02	0.050	1.01	0.98–1.03	0.648
Log PTH (pg/ml)	3.62	0.71–18.39	0.114	0.39	0.02–7.61	0.527
Log NT-proBNP (pg/ml)	4.56	1.43–14.55	0.009	3.38	0.73–15.68	0.113
Hb (g/dL)	1.01	0.78–1.31	0.908			
HT (%)	0.98	0.92–1.04	0.564			
Log PLT (10^3^/uL)	0.25	0.01–5.25	0.368			
RBC (10^6^/uL)	0.96	0.43–2.13	0.933			
MCV (fL)	1.02	0.93–1.12	0.591			
MCH (pg)	1.03	0.78–1.33	0.836			
MCHC (g/dL)	0.90	0.59–1.36	0.621			
RDW > 13.5 %	4.13	1.30–13.13	0.014	3.92	1.05–14.56	0.037
MPV (fL)	1.28	0.81–2.01	0.284			
PDW (fL)	1.14	0.94–1.37	0.180			
PCT (%)	0.22	0.00–126.9	0.631			
P-LCR (%)	1.03	0.97–1.09	0.313			
CRP (mg/L)	1.06	0.97–1.15	0.169			
Albumin (g/dL)	0.88	0.24–3.17	0.840			

*OR* odds ratio, *CI* confidence interval, *eGFR* estimated glomerular filtration rate, *Hb* hemoglobin concentration, *HT* hematocrit, *log NT-proBNP* logarithm of N-terminal pro brain natriuretic peptide, *log PLT* logarithm of platelets, *log PTH* logarithm of parathormone, *MCH* mean corpuscular hemoglobin, *MCHC* mean corpuscular hemoglobin concentration, *MCV* mean corpuscular volume, *MPV* mean platelet volume, *OR* odds ratio, *PCT* plateletcrit, *PDW* platelet distribution width, *P-LCR* platelet-large cell ratio, *RBC* red blood cells, *RDW* red cell distribution width, *CRP* C-reactive protein

Among the examined biochemical parameters, only an increased RDW level was found to be an independent predictive factor for LVDD. Other parameters did not reach statistical significance in multivariate analysis.

## Discussion

Many previous studies have shown that the increased value of RDW level is associated with a worse prognosis for patients with acute (AHF) and chronic heart failure (CHF), with previous myocardial infarcts or strokes [6–10, 19]. Al-Najjar et al. [6] showed that the level of the RDW is important in determining the prognosis among patients with CHF and its prognostic strength is comparable to NT-proBNP levels. The study of Förhécz Z et al. [20] found, however, that the strength of the RDW prognostic in patients with CHF is even greater than the concentration of NT-proBNP. The results of a single study indicate that the RDW is an independent factor of death in patients with pulmonary hypertension and also showed that RDW is statistically stronger and more important than NT-proBNP [11]. In our study, among all laboratory parameters, patients with advanced stage CKD with LVDD compared with patients with advanced stage CKD without LVDD had significantly higher concentrations of RDW level only. Whereas, the level of RDW was not significantly different between early stage CKD patients with and without LVDD. Therefore, the sensitive predict factor for RDW for prognosis LVDD was dependent on the CKD stage.

Importantly, we observed higher RDW levels in patients with eGFR levels <30 ml/min/1.73 m^2^ compared with patients with eGFR ≥30 ml/min/1.73 m^2^, and RDW showed significant negative correlation with eGFR in the studied groups. Additionally, an increased RDW levels can be a novel factor for diagnosis LVDD in this group of patients. Among the examined laboratory factors, the RDW was the strongest prognostic, even more powerful than the NT-proBNP.

However, in the logistic regression analysis, only the RDW was an independent risk factor of diastolic dysfunction among patients with CKD. Other authors showed that the RDW correlated well with ratio of E/Em and NT-proBNP. A value of RDW > 13.6 % and NT-proBNP > 125 pg/ml has high diagnostic accuracy for predicting DHF [13]. In the study of Oh J et al. [21], they found another cut-off value of RDW for predicting E/Em > 15. The value was 13.45 %. In our study, the value of RDW > 13.5 % obtained a good diagnostic value of LV diastolic dysfunction. In another study Solak Y et al. [12], presented the RDW as an independent predictor for endothelial dysfunction in patients with CKD. The mechanisms underlying these relationships remain unclear, although several explanations have been proposed. A direct effect of changes in erythrocyte function on the heart seems plausible, as erythrocytes both carry oxygen to tissues and organs and have an important role in cardiovascular regulation through release of extracellular nucleotides and other mediators [22]. This suggests that the cardiovascular autonomic function could be impaired in patients with high RDW levels. Additionally, an increased RDW level in patients with CHF would be associated with an inflammation. The persistent inflammation is known to be a principal pathophysiologic finding for endothelial dysfunction and heart failure [23, 24]. It has also been suggested that RDW could be a marker of oxidative stress [25], which could be associated with LVDD.

The results of our study are similar to the results of studies available in the literature [13, 21]. They confirm the usefulness of the RDW levels in determining diastolic dysfunction also among patients with CKD. These reports are very important especially when you consider the fact that the RDW is a parameter widely available to clinicians as a result of the morphology of the blood component. The determination of the level of RDW is therefore not associated with additional costs, in contrast to other new diagnostic factors, of which determination is relatively expensive and not always available. The pathophysiological mechanisms are still unclear underlying dependence elevated levels of RDW and LVDD. Therefore, further studies evaluating the LVDD are needed.

There are potential limitations of this study. Firstly, a relatively small, one-center study group. Secondly, we assessed the CRP and serum albumin levels only of 53 patients among of 73 patients enrolled in the our study. Thirdly, we did not measure vitamin B_12_ and foliate levels, and another proinflammatory levels of cytokines, which are one of the potential causes of increased levels of RDW. Fourthly, the lack of blood pressure in our patients.

## Conclusion

RDW values were increased in patients with LVDD in the advanced stage CKD population. Our results suggest that high RDW may be related to LVDD in this group of patients. RDW can be used as an additional factor for the diagnosis of LVDD in patients with advanced stage of CKD.
